# Frontotemporal degeneration (FTD) diagnosis and impact

**DOI:** 10.1093/geroni/igaf122.2522

**Published:** 2025-12-31

**Authors:** Esther Kane, Shana Dodge, Carrie Milliard, Sharon Denny, Penny Dacks

**Affiliations:** Association for Frontotemporal Degeneration, King of Prussia, Pennsylvania, United States; Association for Frontotemporal Degeneration, King of Prussia, Pennsylvania, United States; FTD Disorders Registry, King of Prussia, Pennsylvania, United States; Association for Frontotemporal Degeneration, King of Prussia, Pennsylvania, United States; Association for Frontotemporal Degeneration, King of Prussia, Pennsylvania, United States

## Abstract

Frontotemporal degeneration (FTD), a form of dementia, is the umbrella term for a range of progressive, fatal disorders that include changes in cognition, language, behavior, motor functioning, and personality. FTD is known to be particularly challenging for care partners, as it typically begins earlier than other dementias and has greater financial consequences. There are currently no approved symptomatic or disease-modifying treatments for FTD. The FTD Disorders Registry is a secure online direct-to-participant platform that allows people impacted by FTD to participate in research remotely and provides critical lived experience data to researchers. Registry enrollees, including 956 spouses/partners, family members, caregivers and friends completed the Disease Impact Survey. Nearly a third described it taking more than two years for their loved one to receive their FTD diagnosis and 31% reported that the diagnosing doctor did not explain the diagnosis. Respondents noted symptom impacts on the diagnosed person’s ability to manage finances, run errands, drive or navigate transportation, prepare meals, keep track of appointments, and take their medication. They also endorsed how the FTD diagnosis had impacted their own lives, including altered relationships (81%), increased stress (73%), disrupted plans for the future (72%), and social isolation (48%). While there is a significant unmet medical need for people diagnosed with FTD, it is important for healthcare providers to recognize that FTD care partners also require significant support in navigating this difficult and stressful diagnosis.

